# 

**Published:** 1888-06

**Authors:** 


### BOOK NOTICES.

THE CENTURY grows more and more interesting in its Biography of Lincoln. We have taken the liberty to lay before our readers some points of value from Prof. Atwater's article on Foods and Beverages in the May number.

Our thanks are due for a number of Medical Publications, including :

PROCEEDINGS OF THE STATE SANITARY CONVENTION ; Penn., Benjamin Lee, Sec.

ALIENEST AND NEUROTOLOGIST ; quarterly, St. Louis, Aptil 1888, vol. ix.

